# Identification of kinectin as a novel Behçet's disease autoantigen

**DOI:** 10.1186/ar1798

**Published:** 2005-07-27

**Authors:** Yu Lu, Ping Ye, Shun-le Chen, Eng M Tan, Edward KL Chan

**Affiliations:** 1 Department of Molecular and Experimental Medicine, The Scripps Research Institute, La Jolla, CA, USA; 2 Department of Rheumatology, Shanghai Ren Ji Hospital affiliated to Shanghai Second Medical University, Shanghai, China; 3 Department of Oral Biology, University of Florida, Gainesville, FL, USA

## Abstract

There has been some evidence that Behçet's disease (BD) has a significant autoimmune component but the molecular identity of putative autoantigens has not been well characterized. In the initial analysis of the autoantibody profile in 39 Chinese BD patients, autoantibodies to cellular proteins were uncovered in 23% as determined by immunoblotting. We have now identified one of the major autoantibody specificities using expression cloning. Serum from a BD patient was used as a probe to immunoscreen a λZAP expression cDNA library. Candidate autoantigen cDNAs were characterized by direct nucleotide sequencing and their expressed products were examined for reactivity to the entire panel of BD sera using immunoprecipitation. Reactivity was also examined with normal control sera and disease control sera from patients with lupus and Sjögren's syndrome. Six independent candidate clones were isolated from the cDNA library screen and were identified as overlapping partial human kinectin cDNAs. The finding that kinectin was an autoantigen was verified in 9 out of 39 (23%) BD patient sera by immunoprecipitation of the *in vitro *translation products. Sera from controls showed no reactivity. The significance of kinectin as a participant in autoimmune pathogenesis in BD and the potential use of autoantibody to kinectin in serodiagnostics are discussed.

## Introduction

Behçet's disease (BD) is a systemic vasculitic disease typified by a triad of symptoms including recurrent oral ulcers, genital ulcers and uveitis. In addition, skin, joint, large vessels, nervous system and gastrointestinal systems may be involved. BD is a global disease but has the highest prevalence in the region along the ancient 'Silk Road' in China. The etiopathogenesis of the disease remains unclear but microbial agent triggers, environmental factors, genetic predisposition, neutrophil hyperfunction, endothelial cell dysfunction and immunological abnormalities involving both T and B cells have been implicated. Increasing amounts of research evidence supports the possibility that it is an immune-mediated vasculitis, and that abnormal T-cell and B-cell reactions and autoantigen-driven autoimmunity play pivotal roles [1]. Systemic lupus erythematosus (SLE) is the prototypic systemic autoimmune rheumatic disease with autoantibodies against cellular (particularly nuclear) antigens, some of which are critically implicated in the autoimmune pathology while others provide valuable serodiagnostic markers for the disease. Unlike the picture in SLE and other related rheumatic diseases, in BD, antinuclear antibodies and antibodies to neutrophil cytoplasmic antigens etc. are not present. To date, since neither a specific autoantibody nor pathognomonic pathological index is available to help establish the diagnosis of BD, it is largely or solely based on clinical manifestations [2], and a dilemma in diagnosis is not a rare occurrence in clinical practice. Nevertheless, since the 1960s, there have been reports of autoantibodies against certain unknown components of human oral mucosa in sera of patients with BD. Since then, sporadic reports on findings of autoantibodies in this disease have been described, such as antibodies to retinal antigen(s), heat shock protein (HSP) of some strains of *Streptococcus sanguis *cross-reactive with human HSP polypeptide [3], antibodies to endothelial cell antigens (AECA) and antibodies to α-tropomyosin [4,5], attesting to the complicated humoral immune disorders in this disease.

This investigation was aimed at defining target cellular autoantigens using time-tested and well-established molecular techniques. Immunoscreening of expression libraries using BD sera was used since this approach has been successfully employed in the characterization of many clinically relevant antigens in systemic rheumatic diseases such as SS-A/Ro [6-9] and SS-B/La [10] antigens in Sjögren's syndrome (SjS) and centromere antigen CENP-B [11] in scleroderma. In addition, we have been successful in using this strategy to identify interesting autoantigens that have other biological significance. Examples of these include NOR90/hUBF [12], p80-coilin [13], Golgi autoantigens [14-16] and, more recently, GW182 [17].

## Materials and methods

### Patients and sera

The currently used empirical criteria for the diagnosis of BD in this study were the criteria proposed by the International Study Group for BD (abbreviated as 'International Criteria') [2]. The study subjects of 39 Chinese BD patients comprised 17 males and 22 females, mean age 37 ± 11.3 years old, who were divided into two subgroups: 25 typical BD patients (Group I, satisfying the International Criteria) and 14 clinically diagnosed BD patients who had recurrent oral ulcers and one of the symptoms of genital ulcers, eye symptoms or skin lesions as defined by the International Criteria, as well as additional symptom(s) closely related to BD as listed in the International Criteria, that is, gastrointestinal ulcerations, deep vein thrombosis or arthralgia/arthritis without evidence that the latter symptoms might be related to any other disease (Group II, defined as 'probable BD' in this study). Disease controls included 10 patients with SLE and 10 with SjS, all satisfying corresponding international classification criteria. All BD patients and disease controls involved in the study were patients treated at the Rheumatology Department of Ren Ji Hospital, Shanghai, China, where their clinical data and serum samples were collected. Twenty normal control sera were randomly selected from healthy blood donors working in the same hospital. This study was approved by the institution review board of Ren Ji Hospital which is affiliated with Shanghai Second Medical University, and each patient involved gave informed consent. All serum samples were preserved at -20°C or -70°C until use.

### Cell lines and cell extracts

HeLa (ATCC CCL 2.2) and T24 (human transitional cell bladder carcinoma) were obtained from the American Type Culture Collection (Rockville, MD, USA). A bovine aortic endothelial cell line was kindly provided by Dr Eugene G Levin from the Scripps Research Institute (La Jolla, CA, USA). Cells were cultured in DMEM containing 10% calf serum, harvested and extracted in Buffer A (150 mM NaCl, 10 mM Tris-HCl, pH7.2, 0.5% Nonidet P-40) with protease inhibitor (Complete™; Boehringer Mannheim, Indianapolis, IN, USA). For the preparation of whole cell extract, 10 volumes of Laemmli gel sample buffer [18] were added to the cell pellet, boiled for 3 min and stored at -20°C until use.

### Western blot

Whole cell lysates from bovine aortic endothelial cell, HeLa and T24 cells were resolved individually by discontinuous 7.5% gel SDS-PAGE according to Laemmli's method [18]. Immunoblotting was performed as described by Towbin *et al. *[19] with modifications. Nitrocellulose strips were blocked with 3% nonfat milk in PBS containing 0.05% Tween-20 (PBS-T) and then incubated with BD patient sera and normal control sera (1:100 dilution) at room temperature for 1 h. Filters were washed extensively with PBS-T to remove any unbound antibodies. Bound antibodies were detected with polyvalent, peroxidase-conjugated goat anti-human Ig and visualized by incubating the nitrocellulose strips in chemiluminescent reagents (NEN Life Science Products Inc., Boston, MA, USA) and exposing to Kodak XAR-5 films.

### Screening of phage cDNA expression library with antibody probes

Serum from a BD patient showing the highest antibody titer in immunoblotting was selected as a probe and used at a dilution of 1:300 for initial immunoscreening of approximately 10^6 ^recombinants from a T24 cDNA expression library. The latter was constructed in λZAPExpress vector (Stratagene, La Jolla, CA, USA) and screened as previously described [20-22]. All screenings were performed on duplicate isopropyl β-D-thiogalactoside (IPTG) pre-impregnated nitrocellulose filters, and immunoreactive clones were detected by chemiluminescence. Positive phages were subsequently plaque purified to 100% by two repeated rounds of screening at low plaque densities. Before screening the cDNA library, the BD serum was extensively adsorbed against bacteria and wild-type λZAP phage mixture to reduce background binding.

### Analysis of candidate cDNAs

Purified candidate plaques were subcloned *in vivo *into pBK-CMV plasmids using ExAssist™ helper phage as recommended in the manufacturer's instructions (Stratagene). The recombinant pBK-CMV plasmids were then purified using QIAprep Spin Minprep Kit (Qiagen, Valencia, CA, USA). Restriction enzyme digestion of plasmids with EcoRI and XhoI and electrophoresis in a standard 1.0% agarose gel was used to analyze the length of cDNA insert of each candidate plasmid. The complete nucleotide sequence was determined using Bigdye terminator sequencing and a semi-automated sequencer model 377 (ABI, Foster City, CA, USA). Both nucleotide and deduced amino acid sequences were analyzed for similarity with known sequences using BLAST search [23] and ExPASy Proteomics tools . Secondary structure analysis for coiled-coil motifs was conducted with the software program COILS [24].

### Immunoprecipitation of *in vitro *translation products

Candidate cDNA clones were used as templates for *in vitro *transcription and translation and the products were used as substrates for immunoprecipitation to confirm the specificity of reaction with BD sera. In brief, 1 μg of the pBK-CMV plasmid identified in the screening outlined above was added as template in a 50-μl reaction for the coupled *in vitro *transcription and translation reaction with a rabbit reticulocyte lysate system (Promega, Madison, WI, USA) in the presence of ^35^S-methionine (Trans-^35^S label; ICN Biochemicals, Costa Mesa, CA, USA) and RNasin^® ^Ribonuclease Inhibitor (Stratagene) as recommended by the manufacturer (Promega). Translation was carried out at 30°C for 1.5 h. Products were analyzed in a 12.5% gel SDS-PAGE and stored at -80°C for further immunoprecipitation analysis. The *in vitro *translation proteins were examined for reactivity by sera using immunoprecipitation described [8,25].

## Results and discussion

### Autoantibody detection in sera from BD patients

**Figure 1:**
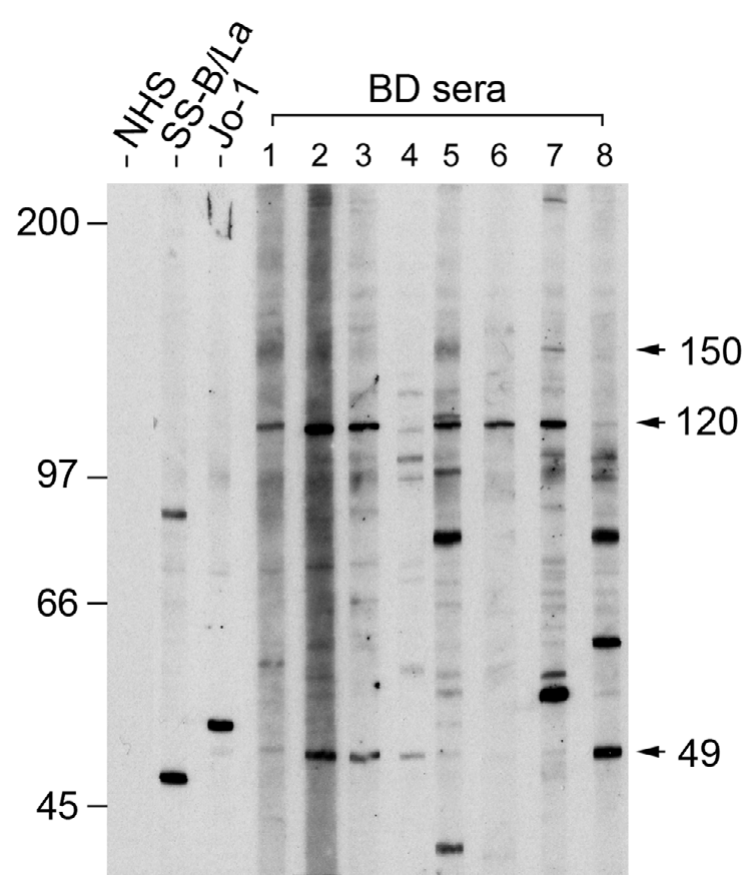
Western blot analysis of autoantibodies in selected BD sera. Whole cell extracts from bovine aortic endothelial cells were separated by a 7.5% gel SDS-PAGE, transferred to nitrocellulose and probed with serum dilutions of 1:100. Several sera showed reactivity to proteins of 49, 120 and 150 kDa. The serum in lane 2 with the strongest reactivity to the 49 kDa and 120 kDa bands was selected as the probe for the expression library screening. Molecular mass markers are shown on the left. NHS represents the normal control. BD, Behçet's disease.

Initial examination of a group of 39 BD patients using indirect immunofluorescence (IIF) on a HEp-2 cell substrate did not yield any characteristic nuclear or cytoplasmic staining patterns. BD is thought by some to be a vasculitic disease involving pathophysiology of endothelial cells, and antibody to endothelial cell antigen (AECA) has been reported. Reports on the prevalence of AECA have varied largely and alpha-enolase was reported as one of the putative target antigens [26]. In this study, the use of bovine aortic endothelial cells as substrate for IIF did not provide any additional data. However, Western blot analysis of the BD sera began to show some interesting autoreactivity using cell lysates from both HeLa and bovine aortic endothelial cells. HeLa cells were initially used for this analysis because they are commonly used in the laboratory as Western blot substrate. Fig. 1 illustrates the common reactivity to 49 kDa and 120 kDa proteins in the endothelial cell lysates. These antigens were also detected in HeLa and T24 cells; the latter cell line was analyzed because our laboratory at The Scripps Research Institute has produced an excellent expression cDNA library from the T24 line and the positive result with the T24 cell extracts allowed us to screen the T24 library. Ig isotype analysis showed that all reactivity was largely IgG antibodies. Since the 49 kDa and 120 kDa bands were observed in cell extracts from bovine as well as human cell lines, these autoantigens might be evolutionarily conserved.

In total, nine out of 39 BD sera (23%) had autoantibody to the 49 kDa antigen and eight (20%) to the 120 kDa antigen. Four BD sera (10%) reacted with both proteins. Additionally, sera that showed common reactivity to the 120 kDa protein also demonstrated a common band that migrated at ~150 kDa, although it appeared weaker than the 120 kDa band. These antigens appeared to have different molecular weights than those of the known autoantigens in systemic rheumatic diseases. In addition, other reactive bands were detected but they were not as commonly shared as the 49 kDa and 120 kDa bands. The 49 kDa protein was shown to be distinct from 48 kDa SS-B/La or 50 kDa Jo-1 proteins (Fig. 1). The 120 kDa antigen was also shown to migrate differently from alanyl tRNA synthetase in another Western blot analysis (data not shown) and did not share any apparent crossreactive epitopes with the 49 kDa antigen. Western blot analyses of 20 normal control sera did not show the reactivities observed with BD sera. In order to further characterize these autoreactivities, a serum sample from the Group I definitive BD patients with the strongest reactivity to 49 kDa and 120 kDa antigens (Fig. 1, lane 2) was selected as antibody probe for expression library screening.

### Kinectin identified as a novel BD autoantigen

**Figure 2:**
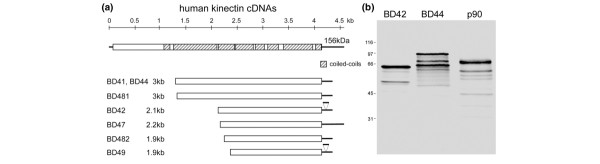
cDNA clones obtained from expression library screen using a BD serum. **(a) **Schematic diagram of the six overlapping cDNAs aligned with published human full-length kinectin sequence (4,816 bp, GenBank accession number NM_182926, shown on top). The open box represents the coding region of the full-length kinectin protein of ~156 kDa. cDNA inserts from the six independent clones BD41/BD44, BD481, BD42, BD47, BD482 and BD49 represent N-terminal truncations and are predicted as coiled-coil domains (hatched). ▽ represents short sequences derived from alternative mRNA splicing in the 3'-untranslated region. **(b) **Autoradiography of *in vitro *transcription and translation products of the candidate clones BD44 and BD42 labeled by [^35^S]-methionine and analyzed on a 12.5% gel SDS-PAGE. Products of BD44 and BD42 gave major bands with the highest molecular mass of 95 kDa and 60 kDa, respectively. The lane marked as p90 represents an unrelated autoantigen used as a positive control for the *in vitro *translation reaction. Molecular markers are shown on the left. BD, Behçet's disease.

After screening 500,000 clones from the T24 cell λZAPExpress expression library, seven immunoreactive clones were isolated and plaque purified in two to three rounds to achieve 100% homogeneity. The cDNA inserts were subcloned *in vivo *into pBK-CMV plasmids, analyzed by restriction digestion using EcoRI and XhoI enzymes, and submitted to direct nucleotide sequencing across the polylinker arms. The cDNA inserts represented six independent clones designated BD41 (identical to BD44), BD481, BD42, BD47, BD482 and BD49. Their identities were established as overlapping partial cDNAs of human kinectin, ranging from 1.9 kb to 3 kb (Fig. 2a). The full-length human kinectin (GenBank accession number NM_182926[27]) has 4,816 bases containing an open reading frame coding 1,357 amino acid residues with molecular mass 156 kDa. All six cDNAs lacked the 5' portion of the kinectin sequence to different degrees but spanned a sequence of kinectin that extended to the 3'-untranslated region. Secondary structure analysis of kinectin protein using the program COILS identified a long region of α-helical coiled-coil domain that extended from amino acid residue 327 to the C-terminus (Fig. 2a, hatched boxes). *In vitro *coupled transcription and translation of BD44 and BD42 clones directed the synthesis of [^35^S]-methionine-labeled polypeptides that migrated at 95 and 60 kDa, respectively, in addition to smaller polypeptides (Fig. 2b). These products had predicted molecular weights of 103 kDa and 75 kDa.

Kinectin was initially identified in chick embryo brain microsome as an integral membrane protein anchored in endoplasmic reticulum and involved in kinesin-driven vesicle motility along microtubules [28,29]. Kinectin consists of a 120-kDa and a 160-kDa polypeptide interacting through the α-helical coiled-coil domain to form a heterodimer [30]. The full-length kinectin is the 160 kDa polypeptide containing an N-terminal transmembrane helix followed by a bipartite nuclear localization sequence and two C-terminal leucine zipper motifs. We presume that the 120 kDa polypeptide detected in Western blot (Fig. 1) is the truncated version of the 160-kDa polypeptide, lacking the N-terminal first 232 amino acids [30]. The N-terminus of the 160-kDa polypeptide consists of a transmembrane domain that anchors kinectin to endoplasmic reticulum [30,31]. This 120 kDa polypeptide is probably the predominant form detected in the Western blot analysis (Fig. 1) because of its preferential solubility due to the omission of the N-terminal transmembrane domain.

Other functions for kinectin have been reported. Yeast two-hybrid screen studies from several laboratories have revealed the interaction of the Rho family of GTPase with kinectin, and have shown the functional links among RhoG, kinectin and kinesin, with kinectin as a key effector of RhoG microtubule-dependent cellular activity [32]. Kinectin was also identified as an important constituent of integrin-based adhesion complexes, which link integrins to the cytoskeleton and recruit signaling molecules [33]. A new study reported that a kinectin isoform lacking a major portion of the kinesin-binding domain is very probably the most conservative form of kinectin; it does not bind kinesin but act as a membrane anchor for the translation elongation factor-1 delta in the endoplasmic reticulum [34].

### Prevalence and specificity of anti-kinectin autoantibodies

**Figure 3:**
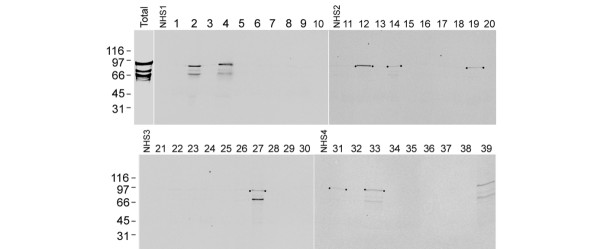
Immunoprecipitation analysis of the 39 BD patients for autoantibody to kinectin. *In vitro *translation products of kinectin cDNA BD44 template was used as the substrate. Nine out of the 39 BD patient sera (23%, lanes 1–39) immunoprecipitated the BD44 translation products. Molecular weight standards are shown on the left. The lane marked 'Total' shows the labeled translation product alone for comparison. Lanes NHS1, NHS2, NHS3, NHS4 are the four normal controls. BD, Behçet's disease.

The *in vitro *[^35^S]-methionine-labeled translation product of BD44, representing the largest recombinant kinectin fragment available, was used as the antigen substrate in an immunoprecipitation assay. Out of 39 BD patient sera, nine (23%) recognized the BD44 translation product (Fig. 3), whereas sera from 20 normal controls, 10 SLE and 10 SjS patients did not show reactivity. Among the nine anti-kinectin positive patients, six (6/25, 24%) were from Group I (definitive BD) including the BD patient whose serum was used in the immunoscreening of expression cDNA library, and three (3/14, 21.4%) patients were from the Group II (probable BD) in this study. According to the Fisher Exact Probability calculation (*P *= 1.00), there is no statistically significant difference for antibody to kinectin between the two groups. The combined data substantiated the finding that kinectin is an autoantigen that can be recognized by sera from 23% of Chinese BD patients in this study with at least one immunoreactive region or autoepitope residing within the BD44 encoded polypeptide.

Currently, there are more than six diagnostic/classification criteria for BD, among which the International Criteria have been applied most extensively due to its relatively high sensitivity (91%) and specificity (96%) [2]. As discussed above, differential diagnosis of BD might be confusing in clinical practice since no specific laboratory test is available, and some patients may have symptoms and signs strongly suggestive of BD but do not fully satisfy the International Criteria, as in the Group II (probable BD) patients in our study group. A number of investigators have pointed out that a comprehensive analysis of the clinical data for a given patient is very important for correct clinical diagnosis of BD, and that classification/diagnosis criteria, including the International Criteria, should be followed but should not be exclusive. The observation that three out of 14 patients in the probable BD group also had antibody to kinectin and the similar percentage of positive reactors between this group and Group I (21.4% versus 24%) supports this notion. The further use of non-clinical parameters such as immunological biomarkers as adjuncts to identify BD patients could be of help in the classification of this disease entity

While our work was ongoing, anti-kinectin antibodies were reported in sera from patients with hepatocellular carcinoma (HCC) [35,36] and aplastic anemia [37,38]. The first HCC report [35] identified kinectin as a tumor-associated antigen from the screening of an autologous cDNA library constructed from the cancer of a 30-year-old patient from Guangxi, China. This report stated that four out of five HCC patients tested were positive for anti-kinectin antibody [35]. In 2004, another laboratory also reported the cloning of kinectin as a tumor-associated antigen from a (presumably) different 30-year-old Chinese HCC patient [36]. In contrast, anti-kinectin antibodies were not detected in other studies of HCC patients associated with our laboratory [39,40]. The reports of anti-kinectin antibodies in aplastic anemia are also very interesting [37,38]. The initial report by Hirano *et al. *identified kinectin by screening an aplastic anemia patient for candidate antigens using a Clontech human fetal liver cDNA expression library and it was concluded that seven out of 18 aplastic anemia patients were positive for anti-kinectin while none of the normal or disease controls had this antibody [37]. In their recent report, Hirano *et al. *reported that anti-kinectin antibodies were found in 39% of aplastic anemia patients from the United States but only in three out of 30 (10%) cases in Japan [38]. In our study reported here, kinectin antibodies were only detected in BD patients and not in normal controls and SLE and SjS disease controls. None of the BD patients with anti-kinectin had signs of HCC or aplastic anemia at the time of diagnosis and at up to 4 years of follow-up. Mapping of epitope(s) recognized by anti-kinectin antibodies may shed light on the question of whether different autoepitopes reside within the kinectin molecule recognized by sera from different diseases.

### Kinectin – a new member of coiled-coil cytoplasmic autoantigens

We have recently reviewed the literature on the growing number of cytoplasmic autoantigens rich in α-helical coiled-coil domains as typified from our study of Golgi autoantigens [41]. Golgi autoantigens are generally high molecular weight proteins between 100 and 350 kDa and rich in coiled-coil domains in the central region with non-coiled-coil or globular domains at both N and C termini. Golgi autoantigens are displayed on the cytoplasmic face of the Golgi complex and are not localized to apoptotic blebs during apoptosis [42]. Giantin, the highest molecular weight Golgi autoantigen reported, is the predominant target of human anti-Golgi complex antibodies and multiple non-cross-reactive epitopes have been mapped spanning the 350 kDa protein [43]. Other high molecular weight autoantigens with similar features have been reported in cytoplasmic and mitotic organelles suggesting that these selected proteins become autoimmunogenic based on their subcellular association and molecular features [41]. For example, in the endosomal compartment, the two known autoantigens are early endosomal protein EEA1 (180 kDa) [44] and CLIP-170 (170 kDa) [45]. There is also a series of centrosomal autoantigens identified as coiled-coil-rich proteins including pericentrin, a 220 kDa protein [46], ninein, a protein with alternatively spliced products of 245 and 249 kDa [47], Cep250 (250 kDa) and Cep110 (110 kDa) [48]. Centromere autoantigens have been described but the two interesting ones related to this discussion are CENP-E [49] and CENP-F [50]; both are high molecular weight proteins (312 to 400 kDa) and have the same type of overall structure as discussed above. NuMA is another large coiled-coil protein located at the mitotic spindle pole and is the most common target autoantigen in sera with mitotic spindle apparatus staining [51]. Non-muscle myosin (~200 kDa) is a cytoskeletal autoantigen [52] that falls in the same group of high molecular weight and coiled-coil-rich autoantigens. These endosomal, centrosomal, mitotic apparatus and intracellular autoantigens are, like the golgins, proteins with high molecular weights and an overall high content of coiled-coil domains. The combination of these two physical features in autoantigens may contribute to the induction and production of autoimmune antibodies in certain disease states. Kinectin is an integral membrane protein largely confined to the endoplasmic reticulum [28,31] and it fits into this new category of autoantigens that are large coiled-coil rich proteins (≥100 kDa) in the cytoplasm.

## Conclusion

Here we report the detection of kinectin autoantibody in 23% of Chinese patients with BD. The identity of kinectin as a BD-related autoantigen has not been reported to date. Autoantibody reaction against kinectin in BD observed in this study further confirms the autoimmune involvement in BD and may provide new inroads into elucidating the immunopathogenesis of the disease. In an effort to clarify the association of BD with antibody to kinectin, it is essential to measure antibody to kinectin in larger patient populations including both BD, probable BD and important autoimmune rheumatic diseases such as SLE, SjS, rheumatoid arthritis etc., as well as those diseases not easily differentiated from BD, such as recurrent aphthous oral ulcer, Reiter's syndrome, inflammatory bowel diseases etc. On the other hand, further analysis of the association of anti-kinectin antibody with different manifestations or disease 'subtypes' of BD is another important project. Anti-kinectin is clearly only one of the antigen-antibody systems identified because there were many other antibodies observed in the Western blot analysis of BD sera. Using other sera for immunoscreening would probably lead to the identification of other potentially important antigen-antibody systems.

## Abbreviations

AECA = antibody to endothelial cell antigen; BD = Behçet's disease; DMEM = Dulbecco's modified Eagle's medium; HCC = hepatocellular carcinoma; HSP = heat shock protein; IIF = indirect immunofluorescence; PBS = phosphate buffered saline; SjS = Sjögren's syndrome; SLE = systemic lupus erythematosus.

## Competing interests

The authors declare that they have no competing interests.

## Authors' contributions

YL performed the study and drafted the manuscript. PY provided technical help throughout the study. SLC and EMT conceived the study, participated in the design and helped in the analysis of the data. EKLC participated in the design of the study, interpreted data and helped to draft the manuscript. All authors read and approved the final manuscript.
